# Supplementary material for: Antibiotic Resistance and Genetic Variability of Acinetobacter spp. from Wastewater Treatment Plant in Kokšov-Bakša (Košice, Slovakia)
Source: Microorganisms. 2023 Mar 25;11(4):840. doi: 10.3390/microorganisms11040840 (PMC10143558; doi:10.3390/microorganisms11040840)
Supplement: Supplementary file 1 [file microorganisms-11-00840-s001.zip › FigureS2.pdf]

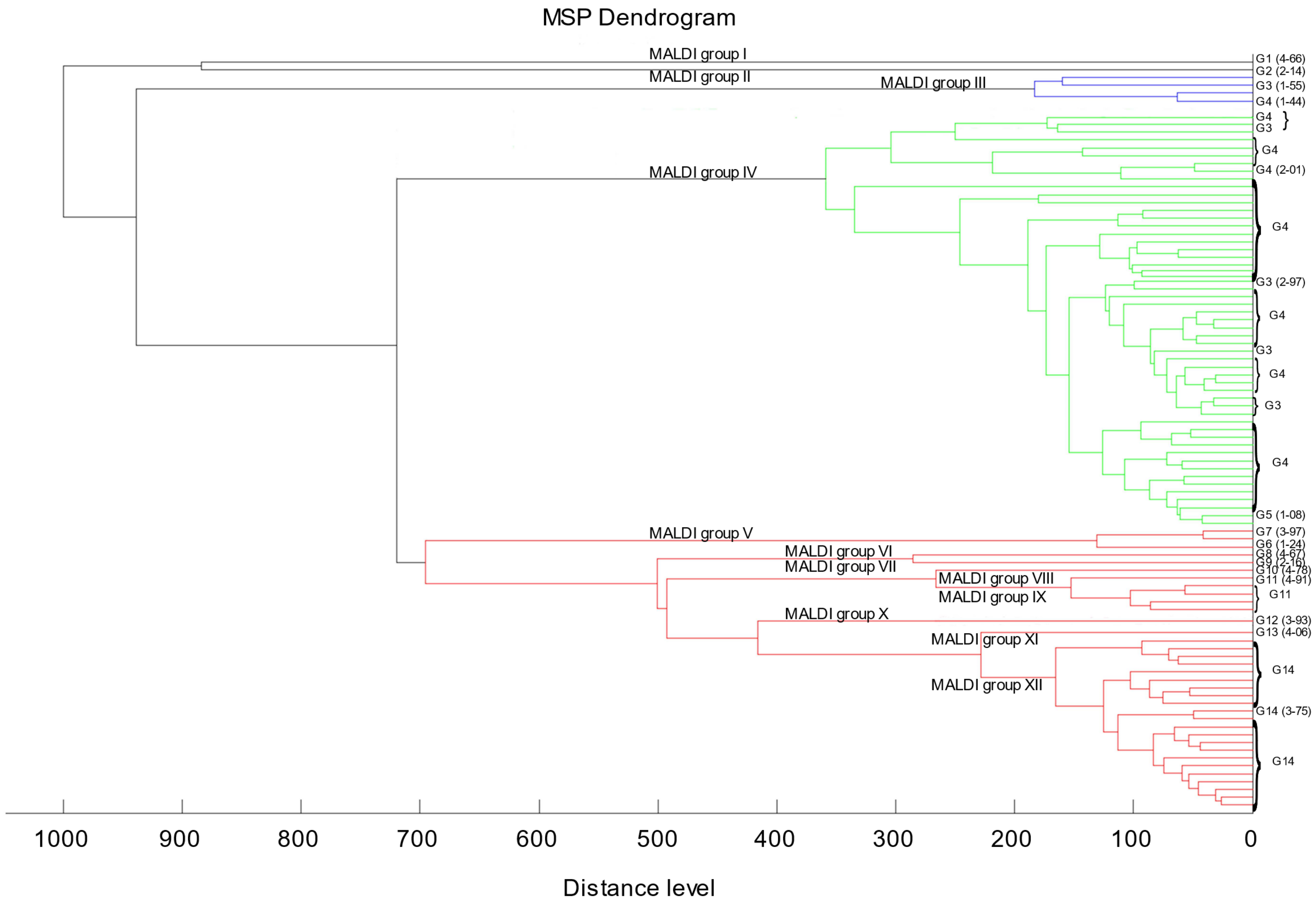

Figure S2. MSP dendrogram created based on the MALDI-TOF MS specific-protein spectra of *Acinetobacter* spp.
G1 - G14 - genotypes obtained based on digestion of 16S rRNA gene using three different restriction enzymes AluI, MspI and RsaI. The dendrogram was generated by MALDI Biotyper 3.0 software with distance displayed in relative units and normalized to a maximal value of 1000.
