# Supplementary material for: Antibiotic Resistance and Genetic Variability of Acinetobacter spp. from Wastewater Treatment Plant in Kokšov-Bakša (Košice, Slovakia)
Source: Microorganisms. 2023 Mar 25;11(4):840. doi: 10.3390/microorganisms11040840 (PMC10143558; doi:10.3390/microorganisms11040840)
Supplement: Supplementary file 1 [file microorganisms-11-00840-s001.zip › FigureS3.pdf]

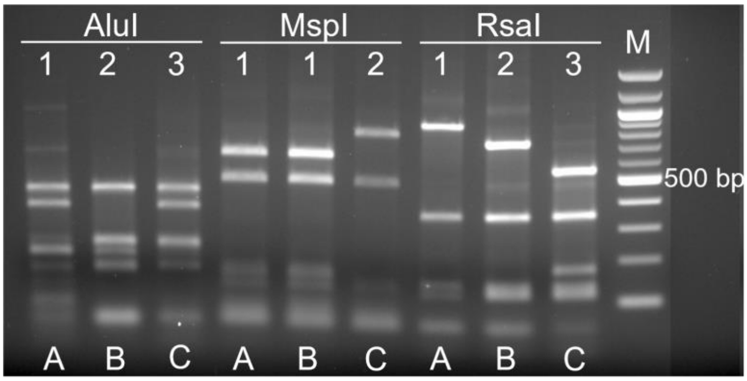

Figure S3. Demonstration of restriction patterns obtained after digestion of 16S rRNA gene of three different isolates (A, B and C).

Numbers in lanes (1, 2, 3) indicate the type of restriction pattern obtained after digestion with the restriction enzyme AluI, MspI and RsaI, M – 100 bp ladder.
