# Supplementary material for: Antibiotic Resistance and Genetic Variability of Acinetobacter spp. from Wastewater Treatment Plant in Kokšov-Bakša (Košice, Slovakia)
Source: Microorganisms. 2023 Mar 25;11(4):840. doi: 10.3390/microorganisms11040840 (PMC10143558; doi:10.3390/microorganisms11040840)
Supplement: Supplementary file 1 [file microorganisms-11-00840-s001.zip › TableS1.pdf]

Table S1. The physicochemical characteristics of the wastewater at the time of sampling on April 10, 2018 (Eastern Slovakia Water Utility Services Company in Košice, Slovakia).

Physicochemical parameter	Inflow	Outflow
Temperature (°C)	11.9	11.8
pH	7.5	7.46
Biological oxygen demand (mg L^{-1})	251	1.7
Chemical oxygen demand (mg L^{-1})	519	< 5
Total dissolved solids (mg L^{-1})	348	< 2
Suspended solids (mg L^{-1})	636	469
Total nitrogen (N) (mg L^{-1})	56	6.31
Organic nitrogen (N_{org}) (mg L^{-1})	28.9	1.46
Nitrite (NO_2-N) (mg L^{-1})	0.005	0.09
Nitrate (NO_3-N) (mg L^{-1})	1	3.05
Ammonium nitrogen (NH_4-N) (mg L^{-1})	26.1	1.71
Total phosphorus (P) (mg L^{-1})	4.48	0.3
Phosphate (PO_4-P) (mg L^{-1})	2.65	0.24
Anionic surfactants (mg L^{-1})	6.19	0.24
Petroleum substances (mg L^{-1})	0.50	0.17
