# Supplementary material for: Antibiotic Resistance and Genetic Variability of Acinetobacter spp. from Wastewater Treatment Plant in Kokšov-Bakša (Košice, Slovakia)
Source: Microorganisms. 2023 Mar 25;11(4):840. doi: 10.3390/microorganisms11040840 (PMC10143558; doi:10.3390/microorganisms11040840)
Supplement: Supplementary file 1 [file microorganisms-11-00840-s001.zip › TableS2.pdf]

Table S2. Correlation analysis of the prevalence of antibiotic resistance within the *Acinetobacter* community in the wastewater treatment plant in Kokšov-Bakša (Košice, Slovakia)

Resistance	Ampicillin	Tetracycline	Chloramphenicol	Kanamycin	Ciprofloxacin
Ampicillin	1[a]				
Tetracycline	-0.015	1			
Chloramphenicol	0.084	0	1		
Kanamycin	0.105	0.102	-0.019	1	
Ciprofloxacin	-0.024	0.172	0.206	-0.031	1

[a] Pearson's correlation coefficient.
