# Supplementary figures and images for: Antibiotic Resistance and Genetic Variability of Acinetobacter spp. from Wastewater Treatment Plant in Kokšov-Bakša (Košice, Slovakia)
Source: Microorganisms. 2023 Mar 25;11(4):840. doi: 10.3390/microorganisms11040840 (PMC10143558; doi:10.3390/microorganisms11040840)

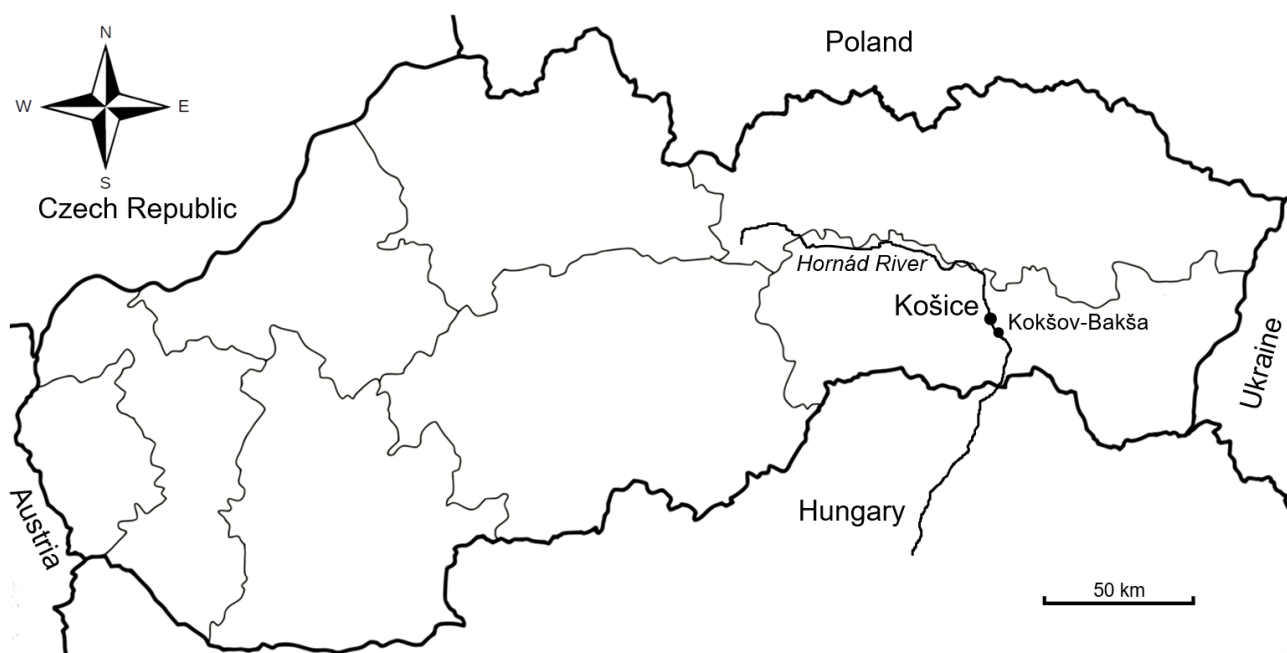

**Figure S1.** Wastewater treatment plant in Kokšov-Bakša near the city Košice (Slovakia).

Supplement: Supplementary file 1 [file microorganisms-11-00840-s001.zip › FigureS1.pdf]
